# A critical discourse analysis of how public participants and their evidence are presented in health impact assessment reports in Wales

**DOI:** 10.1111/hex.12889

**Published:** 2019-04-14

**Authors:** Chris Emmerson, Fiona Wood

**Affiliations:** ^1^ Public Health Wales Cardiff UK; ^2^ School of Medicine Cardiff University Cardiff UK

**Keywords:** community participation, discourse analysis, Health impact assessment, Public Health, qualitative research, sociology

## Abstract

**Background:**

Health impact assessment (HIA) involves assessing in advance how projects affect the health of particular populations. In many countries, HIA has become central to attempts to better integrate health and public participation into policy and decision making. In 2017, HIA gained statutory status in Wales.

This study considers how the public and their evidence are presented within HIA reports and what insights this offers into how public participation is constructed within public health.

**Methods:**

Critical discourse analysis, as described by Fairclough (2003), to analyse seven HIA reports produced in Wales.

**Results:**

Discourses were grouped under four headings. “Consensus and polyphony” relates to the tendency to produce consensus. “Authors and authority” is concerned with how participants and their evidence are shaped by different authorial stances. “Discussions, decisions and planes of action” brings together material on how decision makers are (or are not) brought into contact with evidence in the reports. “Evidence: fragmentation and compression” analyses strategies of abstracting.

**Conclusions:**

This analysis suggests that participants and their evidence are presented in specific ways within HIA reports and that these are particularly shaped by genre, authorial stances and approaches to abstracting and re‐ordering texts. Acknowledging these issues may create opportunities to develop HIA in new directions. Further research to test these conclusions and contribute to a wider “sociology of public health documents” would be of value.

## 1 BACKGROUND

“Impact assessment” as a body of theory and practice emerged in the 1960s to meet a need for formal processes of evidence generation regarding potential environmental hazards related to specific projects.^1^ In subsequent years, formal advance assessment of policy impacts on health has become intertwined with efforts to take a broader approach to health risks to populations and to adopt “health in all policies” approaches.^2^ In an effort to bring consistency to these efforts, now described more generally as “health impact assessment” (HIA), the European Centre for Health Policy published the “Gothenburg Consensus paper” on HIA in 1999.^3^

Government bodies in the UK have increasingly mandated the use of impact assessments in policy development, with statutory requirements for equality impact assessments in Northern Ireland since 1998 as just one example. In Wales, HIA has assumed an increasingly prominent role in health policy since the devolution of health policy decision making to the Welsh Government in 1999. This role has been articulated through a commitment to “embed” HIA within policy and planning processes in Wales,^4^ the creation of the Welsh Health Impact Assessment Support Unit^5^ and legislation within the Public Health (Wales) Act 2017 to provide a statutory basis for HIA.

The Gothenburg Consensus describes HIA as “a combination of procedures, methods and tools by which a policy, programme or project may be judged as to its potential effects on the health of a population”.^3^ ^,p4^ The Consensus follows such influential global health policy documents as the WHO constitution,^6^ the Ottawa Charter for Health Promotion^7^ and the WHO Commission on Social Determinants of Health^8^ in placing a high value on public participation within processes that aim to protect and improve health.

However, Glucker et al^9^ note the lack of a consistently used and/or accepted rationale and definition for public participation in HIA and a considerable body of literature critiquing efforts to “do” public participation in the absence of such a definition. A broader critique is offered by Haigh et al,^10^ who identify a failure to orientate HIA activity within a cohesive and robust ontological, epistemological and methodological paradigm as characteristic of much work described as “HIA”. The objectives of HIA are often described as including elements which are “substantive” (involving the public to improve the quality of decision making) and “normative” (enhancing democratic involvement, often including the empowerment of marginalized communities).^9^ However, it is not clear that these objectives are complementary, with community participation in HIA typically “confused, unreflective and contradictory”.^11^ ^,p230^

Previous analysis of HIA activity and reporting have highlighted ways in which different conceptions of HIA, often reflecting national and regional values, inform HIA^12^ and also considered “downstream” efforts, such as the use of guidelines to make community involvement more representative^5^, ^13^ or ensure that public participation meaningfully contributes to decision‐making.^14^

However, understanding what effective public participation involves may also require looking “upstream” to consider how factors such as asymmetries in information and control of how perspectives are constructed and presented serve actively to create relationships between individuals, groups and their opinions, statements and actions. Power, control and representation are of defining importance in the success of public participation in public health decision making,^15^ and the embedding of health impact assessment within legal and policy processes creates opportunities for broader social shifts in how actors explore the dynamics of power created by HIA, through, for example, its use in community protest.^13^

The specific objectives of this study were to consider how public/community participants in HIA are conceptualized and located within texts, how their voice is heard and what kinds of statements and narratives from community and stakeholder participants are presented as “evidence”.

In addition to these specific objectives, there were more general motivations for carrying out an analysis of how actors, relationships and evidence are presented and positioned in public health texts.

Documents in public health are not simply transparent records of fact, truth or action. They typically represent the culmination of a process that involves commissioning, selecting, drafting, reviewing and publishing (or withholding) material.^16^ These activities themselves involve many influencers and authors, whose beliefs, status, professional and personal affiliations and relationships provide different capabilities and motivations to shape the document, which will then itself influence activities, opinions and decisions. Documents in public health, therefore, both complete, initiate and are themselves social processes. From clinical guidelines to health intelligence reports to patient and public advice to emails and letters to other professionals, documents are one of the main outputs of public health activity. Yet, the sociology of documents appears not to have developed to any degree comparable to the development of sociologies of, for example, primary care^17^, ^18^ or patient safety.^19^

This study therefore attempts not only to engage critically with the representation of public participation and evidence in HIA in order to inform the development of the theory and practice of HIA, but also to initiate and progress wider debate around how these entities are represented in public health more generally.

## 2 METHOD

### 2.1 Analytic approach

Whilst a body of literature based on interviews with participants and considering themes of evidence and public participation in HIA from sociological perspectives is available,^1^, ^12^ there is little evidence of similar material considering the sociology of documents. A database search including the MEDLINE database with all combinations of the terms (a) “health impact assessment,” “HIA,” “Environmental Impact Assessment,” “EIA” and (b) “sociology,” “sociological,” “document analysis,” returned ten results, with no examples of research using general sociological or discourse‐specific methods to analyse HIA documents.

A number of methods of textual analysis for “public” documents have been described in detail in recent years, with critical discourse analysis (CDA) increasingly used to consider how power and legitimacy in health‐related social processes are negotiated and represented in texts.^20^

Critical discourse analysis provides a method for integrating textual analysis at a range of different linguistic and sociological levels and enables analysis of how evidence is described and represented in texts and how different types of evidence are categorized, brought into relationships with each other and assigned value.^21^

The approach to CDA set out by Fairclough^21^ has been used for analysis in this study. CDA involves systematically analysing the use of language and discourses in texts at multiple levels (eg the semantics and structure of sentences; the genres drawn on) to consider how social relations are represented and how those representations might influence the way that world is organized in reality.^21^

Discourses were considered in two particular areas: how participants were located in the text and how their evidence was conceptualized. To ensure a focused approach, three levels of analysis (as defined by Fairclough, 2003)^21^ were chosen for their relevance to the focus of the study: "intertextuality, genre and discourse," "representation of social events" and "semantics and grammar." The study used the checklist of questions provided by Fairclough^21^ for each of these levels to analyse each report. The specific questions are included in the primary analysis, presented online as Appendix S3.

The reports were reviewed to identify sections and subsections which presented material deriving from engagement with “the public”. The definition of "the public" was operationalized as “all those who were included directly in the process of HIA because their health might be affected directly by the changes proposed and also were not included primarily because of any affiliation or expertise deriving from a professional role.”

Each report was reviewed by the first author in terms of each of the questions set out in Appendices S1‐S3, with material organized at the level of these questions within the table. (eg how are participants described?) On the basis of these summaries, broader consistencies and variation within and between texts were identified and discussed amongst both authors. This analysis is available in Appendices S2 and S3.

### 2.2 Selection of texts

The focus of the study was HIA reports completed in Wales. Following discussion with WHIASU, all HIA reports known to WHIASU and in the public domain were listed. To maximize relevance to current debates on HIA, reports were selected starting from the most recent and working backwards. Only one example from any given area of public health was included.

To be included, reports were required to:

- Be explicitly identified as the report of an HIA
- Be publicly available
- Include material derived from direct engagement with individuals who were included primarily because their health might be affected directly by the changes proposed
- Present material in such a way that the contribution of those individuals was distinguishable from other stakeholders who did not meet these criteria

The first seven reports reviewed and considered to meet the inclusion criteria covered the period from 2007 to 2018 The next report that met the criteria was from 2001 and did not reflect contemporary HIA practice in Wales in a number of ways, with methods of involving participants in particular poorly described. The authors then considered whether these seven reports represented a sample that was sufficient to address the research question. Malterud et al's^22^ concept of “information power” was used to address the issue of sample adequacy. “Information power” defines five dimensions that can be used to classify study objectives and determine whether more or less material will be required to address them. Using these dimensions, this study would be considered narrowly rather than broadly focused; sample specificity is narrow (there is a defined set of publicly available texts); the quality of dialogue is good (the authors are typically experienced in carrying out HIAs); theory is being applied rather than developed, and commonalities between reports are sought to a greater degree than examples of divergence. This suggests relatively high “information power,” with seven reports considered sufficient to meet the objectives of the study.

Details of the chosen reports are included in the Results section.

## 3 RESULTS

### 3.1 Details of reports

The seven identified reports are summarized in Appendix S1: Table S1.

Reports are available through the WHIASU website (https://whiasu.publichealthnetwork.cymru/en/hia-reports/). The exception is the Llangefni HIA, which was provided by WHIASU, and is available from the authors.

The reports vary considerably in terms of length, approach taken to public participation and organization of participant material.

Although consideration of the settings and contexts in which the texts were created was outside the scope of this study, it is recognized that factors such as availability of resources including time, personnel and money and pre‐existing links between communities and health‐care organizations will have created opportunities and limitations which have shaped each report in distinctive ways. It is also not clear from the reports how structural constraints, such as the ability to influence the terms of reference of HIA, may have restricted HIA processes. As the analysis describes, the frequent lack of clear connection within the reports between what is written and what is done with what is written makes evaluation of the relationship between HIA reports, HIA processes and subsequent decisions and actions extremely difficult. Full analysis is included online as Appendix S3. Analysis is presented here under two headings, which bring together material related to how participants and their evidence are represented in the reports.

### 3.2 Participation

Three categories of actor consistently emerge as important in considering how participants are located within HIA reports: decision makers, report authors and participants themselves.

Decision makers are rarely if ever positioned as actors in relation to those participating in workshops or focus groups. In most texts, there is no mention at all of who will be making decisions. Where entities involved in decision making do intersect with participants, it is often non‐specifically (“the authorities”) or with the given project rather than a permanent body cited:

One resident wanted to know why the authorities decided on this scheme after new houses had been built in such close proximity [Ffos‐Y‐Fran, p. 56]

The Ffos‐Y‐Fran scheme will remove a further 900 acres of the remaining urban common land from the mountainside [Ffos‐Y‐Fran, p. 65]

The Gaer report brings Derwen (the Housing Association planning the new estate) most clearly into the text as an actor:

Recommendation ‐ Derwen to promote activities already being provided in the Community Centre [Gaer, p. 12]

However, the social processes that link participants and decision makers are absent: how decision makers might act on the insights and recommendations from the participants is never clear.

In most cases, author names and institutional affiliations are provided but authors almost never re‐appear within the text. This “disappearance” conceals the authorial work to select and summarize material, in turn creating a sense of being “closer” to the participants' actual words through submerging any intermediate authorial voice.

Where authors do “re‐appear,” their position in relation to the participants is often ambiguous. In this extract, it is not clear why the authors chose to engage at this point but not at other points in the discussion, nor how this intervention positions them—as impartial assistant? as supporter?—within the process:

one stakeholder [noted]….air pollution would have a long‐term cumulative impact on the population in Splott. One of the facilitators, Nick Hacking from WHIASU, pointed out at this point that Environmental Impact Assessment (EIA) does not measure cumulative impacts. [Splott, p. 31]

The following example also demonstrates ambiguity in apparently reversing the actual order of events within the order of the text so that what is expressed by participants is positioned as driving the actions of the author towards further research:

None of the focus groups were aware of the existence of toilet finding Apps for smart phones. Three of these Apps were examined by the author…. [Anglesey, p. 42]

There are also occasions in which the “the HIA” itself becomes the subject of actions:

The HIA facilitated some interesting conversations about the Housing development's impact [Gaer, p. 9]

The HIA also identifies that the biomass development is likely to have cumulative impacts [Llangefni, p. 3]

In general, “the community” is presented as a collective with no distinct voices or subgroups.

There were only a very small number of reports in which the participants were placed in a social relationship with each other or in which identities other than “community member” or “participant” were acknowledged:

neighbours to help each other with transport, sharing costs, socialising [Gaer, p. 10]

An ex‐ miner attending the workshop stated that to extract approximately 10 m ton of coal the operators will need to also remove approximately 100 m ton of rock and stone [Ffos‐Y‐Fran, p. 37]

There was considerable variation between texts in ways in which voices are presented, ranging from frequent direct quotation (eg Ffos‐Y‐Fran), indirect quotation (eg Splott) to summary notes produced by report authors (eg Llangefni). Direct quotation is typically introduced to support or add emphasis (and possibly legitimacy) to a summary of participant opinions:

Residents attending the workshop believed that the scheme would have a negative effect on inward investment. One resident stated that: “It is clean industries we are looking for, but this will put off those industries.” [Ffos‐Y‐Fran, p. 53]

Cam Ymlaen “wakes you up a bit!” [Cam Ymlaen, p. 3]

Only a very small number of direct or reported quotations are based in the lived experiences of participants, and these are generally brief, usually general (rather than referring to specific events) and appear embedded in discussions of a range of evidence for and against specific proposals:

Could be less stressful I guess. Because always like if you rent a house you always feel this kind of stress. Fear that you will be kicked out if you don't pay the rent, going to be kicked out… [WALLS, p. 58]

You cannot leave your windows open ‐ on a nice day it's nice to leave your windows open. I don't leave my windows open any more. [Ffos‐y‐Fran, p. 39]

Topography can make walking around the area difficult with few benches to sit on [Gaer, p. 11]

Statements were typically future‐ and environment‐orientated and rarely located individuals in the context of the social events that formed the context of their lives as experienced, although it should be noted that there were a small number of specific statements that did draw on direct personal experience:

Got up late, rushed out, so the place was not, what I would call, inviting for anybody to visit. And while I was out at work I got a call from him to say he's at the house repairing the boiler. Well actually that's not acceptable at all. [WALLS, p. 57]

In those reports that draw heavily on the management reporting genre, use of tables and brief summary statements (eg Gaer, Cam Ymlaen) typically puts the reader at a considerable distance from what words might originally have been uttered by re‐arranging a temporal order into a thematic order. These texts also consistently use a neutral register, even when the text that frames or introduces the participant section makes explicit reference to different tones of voice within the discussion:

The workshop followed a systematic process and provoked a lively discussion. [Gaer, p. 19]

These strategies have the effect of “flattening” the emotional register of the report, making it difficult to see which points may have been more or less controversial or strongly felt.

### 3.3 Evidence

Three dominant genres were identified—academic paper, management report and narrative—but these combined and overlapped within reports.

The conventions of the peer‐reviewed academic paper, such as use of references in an academic style (eg Ffos‐Y‐Fran, Anglesey) and descriptions of sampling strategies (eg Anglesey, Gaer), are strongly in evidence in several papers.

A second genre, the management report, incorporates features including short declarative sentences, arrangement of text into two boxed columns and summaries strictly organized into pre‐defined categories (typically “positive” and “negative”) that together suggest a document organized around the needs of time‐pressed decision makers (eg Gaer, Anglesey, WALLS).

The “narrative” genre, presenting an account that follows discussion between participants, usually with reported speech, but offering an apparently detailed, chronological “story,” grounded in the social interactions of the participants comprises a third genre. Typical of this genre are rhetorical devices used to invest the text with qualities more closely associated with fiction than formal writing, such as changing the “pacing” of the text and creating a sense of dialogue, even when speech is reported:

Yet another stakeholder wondered…. [Splott, p. 29]

One stakeholder was quick to point out… [Splott, p. 31]

This “polyphonic” approach (in contrast to other genres, in which “the community” is presented as speaking with a single voice) structures the narrative, with stretches of text in which point is contrasted with counterpoint through multiple voices, before the authors offer a summary:

The first theme to emerge was economic activity…

…One stakeholder said that they felt unsure of Viridor's specific plans for Combined Heat and Power (CHP), but that it might be a good way to attract other businesses to the immediate area around the planned site in Trident Park. Others talked about how CHP could be a major economic benefit to the area … a potential reduction in fuel poverty was regarded as one of the strongest benefits that stakeholders hoped was still on offer. Both workshop facilitators pointed out at this point that there are links between fuel poverty and ill health in the research literature. [Splott, pp. 28‐29]

In this extract, the authors set the scene, appear to “drop back” to allow the voices of participants to “come forward”, then re‐emerge to provide evidence in relation to a point made by the participants. This analysis further illustrates the frequent ambiguities in the authorial role, even within adjacent paragraphs of text.

In terms of what is presented as evidence from public participation, the majority of statements by participants that were directly referred to in all reports concerned expected outcomes of particular plans:

There was therefore a recognition that more waste industry in the area will likely make these potentially negative perceptions of Splott harder to shift in the future [Splott, p. 32]

The workshop was unanimous in its view that reduced availability of facilities would not impact on a family's choice to visit certain areas [Anglesey, p. 38]

A further relatively common category of statement was suggestions for improvement of schemes:

Workshop participants also suggested that support agencies with understanding of the needs of tenants….should be involved in training… [WALLS, p. 35]

Although different categories of evidence were presented within reports, this evidence typically appeared fragmented, with relatively few examples of different types or items of evidence being linked together. Those reports that draw on narrative genres for their structure tend to present more “chains” of evidence, typically using connecting words that suggest a logical flow of argumentation:

Several stakeholders expressed their fears that Viridor's facility would likely add to a picture of already an overly‐industrialised area. This was linked to the previous discussion about concerns for the cumulative effect of adding another source of pollution to Splott ward, however, it went further in terms of the perceptions that outsiders have of Splott. There was therefore a recognition that more waste industry in the area will likely make these potentially negative perceptions of Splott harder to shift in the future. [Splott, pp. 32‐33]

## 4 DISCUSSION

Discourses were grouped under four headings: (a) consensus and polyphony; (b) authors and authority; (c) discussions, decisions and planes of action; and (d) evidence: fragmentation and compression.

### 4.1 Consensus and polyphony

The majority of reports present a summary or set of recommendations as a consensus view of the participant engagement. Only one (WALLS) specifically notes any lack of complete consensus in its summary:

There was no consensus as to whether conditions would improve for the most vulnerable groups [WALLS, p. 7]

However, despite the purpose of the HIA being to evaluate the impact of the WALLS scheme on vulnerable tenants, there is no further discussion of this disagreement, although it is possible that the recommendations within the document, which are presented as emerging as a consensus view, were seen to have addressed this issue through inclusion of proposals for evaluation.

The theory and practice of HIA, whether focused narrowly on supporting effective decisions or broadly on democratic involvement typically recognizes a need for the process to produce consensus of some kind. However, the specific strategies used across these reports through which consensus is produced and presented show distinct patterns of commonality and divergence.

The use of genre and authorial standpoint creates consensus in a range of ways: for example, collapsing of categories of individuals into broader overarching categories and breaking down of discussions into “balanced” positive and negative views from which an author emerges to construct a summary or recommendation.

However, the consensus created through the text appears to take on different qualities dependent on the genres drawn on across the text. The academic paper as a genre typically uses descriptions of process to demonstrate transparency and therefore the legitimacy of the reality that the author makes claim to have discovered.^23^ The HIA reports drawing on this genre therefore not only suggest a single opinion exists, but also that it can be discovered through following a clear process. By contrast, reports that draw on the narrative genre present the development of consensus as a constructive role in which a number of voices are heard, building on each other, until a consensus can be finally articulated by the authors. One effect of bringing the reader “closer” to the voices of the participants through grounding the text in concrete activity of discussion is to suggest legitimacy of the text and its conclusions. In both these genres, it is notable that direct quotation, where used, tends to follow authorial summaries of material, rather than itself introducing or framing authorial perspective.

Analysis of the management report genre highlights a different aspect of consensus. Whilst this genre appears considerably less dependent on producing a single and coherent (but authored) point of view, the radical re‐ordering of material from a temporal to thematic sequence, the flattening or “bracketing off” of emotive content, the presentation of material in formats (such as tables) that reflect decision‐making processes and the frequent avoidance of sentence subjects all suggest strong pressure towards regularity and impersonality. One interpretation of this is that consensus within these texts is not guaranteed by claiming to demonstrate that participants converge on a single view but rather by a claim that all voices are essentially the same. Strategies such as using abstraction to put “distance” between what was said and how it is represented through summary, removing individual voices from their context, stripping emotive context from representations of speech and re‐ordering material all tend to limit competing definitions, privilege authorial standpoints and produce “absolute” language in the form of single, declarative recommendations or summaries.

The narrative genre suggests at least the possibility of representing difference; however, the authorial voice tends to bracket off any divergence of opinion, stressing unanimity but also positioning itself by implication as the “middle ground” of consensus. The academic paper genre and management report genres tend, through their inherent need to produce consensus across voices, to suppress differences in power or norms, also bracketing off difference.

### 4.2 Authors and authority

The authorial role, particularly in relation to participants, varies markedly between reports. In some reports, notably those that drew most heavily on the academic paper genre (eg Gaer), the authorial standpoint appears as relatively consistent, although as discussed above, the “work” that the authors do in shaping the text is largely unacknowledged.

However, in a number of other texts, the authors move from background to foreground and back again in unpredictable ways (eg to “appear” suddenly in the text with a reference in support of a participant point in the Splott report) and/or appear to “take over” the narrative from the participants, sometimes in mid‐sentence (eg Anglesey, Ffos‐Y‐Fran). This ambiguity in the role of the authors appears in more subtle ways, such as using quotation marks to highlight certain words in the text, suggesting that these privileged words have special meaning in bringing the reader closer to the participant.

The variation in tone, authorial distance from the participants and acknowledgement of authorial agency can present the authors in multiple roles—impartial recorder, supporter, “expert witness,” skilled summarizer—within a single text.

Each role, and the authorial work that supports it, has value in presenting the authors in specific ways to specific audiences. For example, appearing as a supporter may be useful in producing consent and consensus in relation to change; adopting the persona of an impartial arbiter may reflect a corporate need to demonstrate transparency and fairness; creating a narrative may make the text more readable and engaging and provide legitimacy through a sense of being “closer” to the participant voices. Whether these roles are compatible in terms of creating a coherent authorial standpoint, or resolvable within the repertoire of genres used, is less clear and it is interesting to note that whilst the pressures of genre and style tend to collapse differences between participants, those same pressures appear to produce a diverse range of roles for authors.

### 4.3 Discussions, decisions and planes of action

The absence or positioning in the abstract of “decision makers” serves to place them on a separate plane to participants. They do not emerge in texts as a constituency with a variety of pressures acting on them and containing a range of opinions. Rather, they appear as an abstract, depersonalized group whose motivations and preferences are unknown and possibly unknowable.

This treatment of decision makers can serve to isolate the participants and their activity as a social process from other elements of the HIA. The lack of context and connection with decision making and decision makers means it is often not clear how statements arise in relation to specific questions or other social processes: there is a sense in which participants appear in the text to be acting in a vacuum. There is little or no discussion of where a given HIA fits within the context of decision making: for example, constraints on methods or resources, how the HIA as either a process or an output is located in relation to decision‐making processes, what impact it is anticipated to have or how that impact might be measured.

Decision making and public participation are both social processes within health impact assessment. Texts describing the processes and outcomes of public participation may benefit from considering how these processes are linked and can be brought in contact with each other in the text.

### 4.4 Evidence: fragmentation and compression

All the reports summarize speech, abstracting it to greater or lesser degrees from its original context, depending largely on the genres employed. The academic paper and management report genres tend to radically “disembed”^24^ material from its origins as context‐dependent speech and “re‐embed” it into a text that presents itself in a single “neutral, objective” register. However, reports drawing to a greater degree on the narrative genre, particularly those using direct quotation (eg Ffos‐Y‐Fran), may include content across a range of emotional registers that is anchored both in lived experience and in the social processes through which it is elicited.

The processes of disembedding and re‐embedding have two notable implications. The first is that isolating statements from their context leads to the breaking of causal chains that serve to strengthen our understanding of “why” and “how” different courses of action might be of value.

The second implication is that the abstraction of evidence from social processes, particularly combined with the compression of tonal register in the reports, may limit our ability to understand how causes and outcomes interact and how participants value them. In those reports where evidence of processes of reasoning and beliefs is suppressed or collapsed into broader categories or processes (eg where whole conversations rather than verbal exchanges are summarized), it becomes difficult to recreate the structure of participant beliefs or preferences: any processes by which individual priorities were negotiated within group discussion, for example, are not available to us to evaluate.

### 4.5 Strengths and weaknesses

Health impact assessment is an activity which produces (in the cases studied) written reports. Whilst we have drawn on various sources that offer guidance on the practice of HIA,^5^, ^10^, ^20^ our focus has been on the reports themselves, rather than processes. Further research to explore discourses concerning the production of HIA would be of value. This study used HIA reports produced and published in Wales. This focus allowed clear strategies for selection and linked analysis to specific principles and practices within a defined space. However, these restrictions may limit generalizability and further research would be of value to establish how applicable this analysis is to HIA in other regions and countries. Further research might also look for evidence on reports that are unpublished or uncompleted and evaluate whether discourses within these reports differ substantially from those presented here.

Analysis was also restricted to language: further research could use multimodal analysis to include elements such as photographs, maps and diagrams.

This study used CDA to consider broad topics related to the representation of the public and their evidence. As with any methodological approach, CDA presents limits as well opportunities, and other approaches (such as ethnography) might provide complementary insights.

Overall, this study attempted to address relatively novel objectives using a method not commonly used in public health research. Further research addressing these (or similar) objectives and/or using these methods in public health research would be of value to assess the significance of our findings.

### 4.6 Recommendations

Given that this study used a relatively novel approach and subject material, further research to develop or challenge the conclusions and to assess their applicability beyond HIA and/or Wales would be of value.

Whilst the goal and objectives of this study were descriptive rather than prescriptive, the analysis also suggests ways in which those engaging in HIA might usefully reflect on and develop their practice. In particular, increased reflexivity from report authors in relation to how they locate themselves in reports and how they allow the voices of participants to “get in,” might produce new approaches to representing participants in reports.

Acknowledgement within the text of the role that decision makers and decision‐making processes play in HIA may create opportunities to link evidence from public participation more directly to the social practices shaping that public participation and how it is used.

More broadly, incorporating analysis of discourses within HIA reports could usefully inform the evaluation of individual HIA projects and the use of HIA generally by a range of stakeholders, both within statutory and/or elected bodies and other organizations, such as activist groups.

The authors hope that the recommendations will support the continued development of public participation in public health decision making in Wales. However, it is also hoped that, taken together and considered in contexts beyond the borders of Wales, these recommendations might also contribute to the creation of the “new knowledge spaces” envisaged for HIA by Elliott and Williams.^1^

## CONFLICT OF INTEREST

No conflict of interest has been declared by the authors.
