# Supplementary material for: A critical discourse analysis of how public participants and their evidence are presented in health impact assessment reports in Wales
Source: Health Expect. 2019 Apr 14;22(3):585–93. doi: 10.1111/hex.12889 (PMC6543135; doi:10.1111/hex.12889)
Supplement: Supplementary file 1 [file HEX-22-585-s001.docx]

**HEX-2018-3035, Public participants’ evidence in HIA**

**Appendices**

**Appendix 1 – Summary of reports included in the analysis**

**Table 1 – Summary of reports included in the analysis.**

| **Report name** | **Published by** | **Theme** | **Method of participatory consultation** | **Publication year** | **Referred to in text as** |
| --- | --- | --- | --- | --- | --- |
| Health Impact Assessment (HIA) of the Gaer Bungalow Estate and new Derwen Development | WHIASU  Derwen  Public Health Wales | Housing | Half day facilitated workshop using framework for discussion based on model of wider determinants of health developed by WHIASU | 2017 | Gaer |
| Comprehensive Health Impact Assessment on Anglesey’s Public Toilet Provision | Isle of Anglesey County Council | Public toilets | Half-day facilitated workshop using framework for discussion based on model of wider determinants of health developed by WHIASU | 2016 | Anglesey |
| Health Impact Assessment of the Welsh Agent and Landlord Licensing Scheme | Welsh Govt | Licensing | Four group and two individual semi-structured interviews | 2014 | WALLS |
| Health Impact Assessment for Llangefni Biomass Plant | Isle of Anglesey County Council | Energy | Facilitated workshop (no further details provided) | 2013 | Llangefni |
| Interim Report of the Health Impact Assessment (HIA) of the Waste Incineration Development Planned in Trident Park, Splott, by Viridor Ltd | WHIASU  Public Health Wales | Waste management | Evening facilitated workshop. Three sessions (impact on vulnerable groups, discussion of development and mitigation of impacts) each introduced by presentation. In addition, attendees provided with pre-event links to newspaper stories and planning papers. | 2010 | Splott |
| Health Impact Assessment of the Cam Ymlaen Project, Conwy | WHIASU | Mental health service | Half-day facilitated workshop, including presentations on work of Cam Ymlaen and framework for discussion based on model of wider determinants of health developed by WHIASU | 2008 | Cam Ymlaen |
| A Report of a Health Impact Assessment Study of an Opencast Scheme at Ffos-Y-Fran, Merthyr Tydfil | Ffos-Y-Fran Health Impact Assessment Steering Group | Open cast mining | One day facilitated workshop, including presentation from Environment Agency Wales and breakout sessions. Also resident survey, local data collected by residents and letters, newspaper articles and other primary and secondary sources | 2007 | Ffos-Y-Fran |

All reports are available through the WHIASU website with the links listed below. The exception is the Llangefni HIA, which was provided by WHIASU and is available from the author.

**Gaer:**

<https://whiasu.publichealthnetwork.cymru/files/9915/0590/8072/Final_HIA_Report_for_the_Gaer_Bungalow_Estate_and_development.pdf>

**Anglesey:**

<https://whiasu.publichealthnetwork.cymru/files/6315/1782/0921/Public_Toilet_HIA_Final.pdf>

**WALLS**:

<https://whiasu.publichealthnetwork.cymru/files/6514/9554/0684/proposed_Wales_Agent_and_Landlord_Licensing_Scheme.pdf>

**Splott**:

<https://whiasu.publichealthnetwork.cymru/files/1414/9554/9969/Splott-Community-HIA-Interim-Report-MASTER-26-5-10.pdf>

**Cam Ymlaen**:

<https://whiasu.publichealthnetwork.cymru/files/6214/9553/7111/Health_Impact_Assessment_of_the_Cam_Ymlaen_Project_Report.doc>

**Ffos-Y-Fran:**

<https://whiasu.publichealthnetwork.cymru/files/7914/9554/0855/FYF_Final_report_June_07.pdf>

**Appendix 2 – questions used to develop analysis**

*[it is expected that, if included, this would be in the form of an online appendix]*

The plan for analysis was developed by translating questions around the representations of ‘participation’ and ‘evidence’, as described in the method, into progressively more focused analytic questions, drawing on question schemes developed by Fairclough (2003). It was recognised that questions organised under one heading might have relevance to points of analysis in the other.

**Participation**

How are public/community participants in HIA conceptualised and located within the text and how is their voice heard?

- At the level of representing social events, how are different actors represented and brought into contact with each other?
  - Which actors are present and which are absent?
  - How are these participants described – e.g. collective/individual nouns
  - Are there points of contact between individuals and/or groups described?
  - Are categories of equivalence and/or difference assumed, established or collapsed?
- At the level of intertextuality, how do the voices of community/public participants ‘get in’ to the text?
  - To what degree is the contribution of the participants ‘dialogized’ – how is their speech translated into the text (e.g. direct reporting, indirect reporting, etc.)?
  - How does this orientate them in relation to difference and consensus?
- At the level of semantics and grammar: how does the use of language give or withhold agency from public/community participants?
  - Are they predominantly described in the active or passive voice?
  - Are personal or impersonal forms used?

**Evidence**

What kinds of statements and narratives from community and stakeholder participants are presented as ‘evidence’ in the text and what are the regularities and differences in presentation within and between texts?

**Analytic approach**

- At the level of discourse, genre and intertextuality: How are statements and narratives organised and brought into relation with each other within the text?
  - What genres are drawn on and what are the implications of these choices?
  - How is the text physically organised on the page and does this create specific discourses?
  - How is ‘evidence’ from community participants brought into contact with other forms of evidence (if at all)?
- At the level of representation of social events: what categories are described/created within the text to define how participants present themselves?
  - What kinds of statements/narratives appear in the text referring to community participants? (e.g. report of experience, identification of problem with proposal, etc.)
  - How are these organised in relation to time and space (do they typically refer to immediate or long-term outcomes? Do they concern themselves with different geographies?)
- At the level of semantics and grammar: how are statements presented in semantic and grammatical terms and what regularities and differences emerge?
  - How are these statements framed in terms of:
    - language register (e.g. emotive / neutral)
    - grammatical features (e.g. declarative / conditional clauses; active/passive mood)
    - semantic relations between clauses (e.g. separated or linked clauses)

**Appendix 3: primary textual analysis**

|  | **Gaer estate** |
| --- | --- |
| At the level of representing social events, how are different actors represented and brought into contact with each other?  *Which actors are present and which are absent?*  *How are these participants described?*  *Are there points of contact between individuals and/or groups described?*  *Are categories of equivalence and/or difference assumed, established or collapsed?* | Initially all participants are listed by job function, community participants identified as “Derwen HA residents from the Gaer Estate” [p9] – residents orientated primarily in relation to Housing Association and secondarily as a geographical community  Categories of difference therefore initially collapsed – there is no distinction between participants  However, different terms then suggest distinct constituencies within community: “neighbours”; “new residents”; “wider community”; “residents”  ‘Vulnerable groups’ identified [p 9/10], but no evidence that they are subsequently distinguished between: collapsed into single category  HIA team noted and named, but HIA described in terms of active entity in itself (“the HIA facilitated….”); individuals not identified with specific actions/words  ‘Derwen’ (housing association) typically described in terms of actions either ongoing or resulting from HIA (“Derwen to promote activities…”)  Other entities described in terms of specific needs (“Additional patients can be accommodated within the GP surgery”)  Derwen as decision makers only occasionally referred to, not brought directly into contact with participants on a social plane – impression is that participants make requests and Derwen responds – the HIA appears as the decision making process  Participants’ individual comments evaluating process presented in appendix |
| At the level of intertextuality, how do the voices of community/public participants ‘get in’ to the text?  *How is their speech translated into the text (e.g. direct reporting, indirect reporting, etc.)?*  *How does this orientate them in relation to difference and consensus?* | No direct or indirect quotation  Text presents ‘specific recommendations from the group’ as indirect reporting alongside summary  Single reference to discussion on specific point [p11], otherwise all statements presented as consensus of single entity of ‘residents’  Not always clear who is speaking – e.g.  “..additional benches on the hill leading to the development would help.  NCC Highways unlikely to accept responsibility to maintain additional benches/seating” [p16] – possible that evaluation of likelihood of NCC Highways accepting responsibility comes from residents, but more likely comes from authors? |
| At the level of semantics and grammar: how does the use of language give or withhold agency from public/community participants?  *Are they predominantly described in the active or passive voice?*  *Are personal or impersonal forms used?* | Residents consistently referred to in third person - e.g. “Some residents have to plan shopping weeks ahead” [p10]; “Isolation for those with mobility issues” [p12]  Single but notable instance of switch between pronouns – “Sense of control over where they live and participation, how to maximise this in the development. Having your own space is important” [p 14]. Not clear who ‘your’ refers to (specific to Gaer residents or wider assumption about individuals). Notable that shift in grammar parallels single instance of shift of focus from practical/specific issues to wider sociological/psychological perspective |
|  |  |
| At the level of discourse*,* genre and intertextuality: How are statements and narratives organised and brought into relation with each other within the text?  *What genres are drawn on and what are the implications of these choices?*  *How is the text physically organised on the page and does this create specific discourses?*  *How is ‘evidence’ from community participants brought into contact with other forms of evidence (if at all)?* | “As statistical evidence, best practice case studies and other robust research on the health impacts of housing had been considered already….” [p9] – other evidence explicitly bracketed off from this discussion  Heavily structured around ‘Positive/Opportunities’ versus ‘Negative/unintended consequences’ – HIA presented here as a decision making tool, balancing evidence with implication that each point is equal of each other point. Also uses categories drawing on Dahlgren and Whitehead (1992) to organise material at different levels of social analysis  Points translated into recommendations with no description of how this transformation occurred |
| At the level of representation of social events: what categories are described/created within the text to define how participants present themselves/their evidence?  *What kinds of statements/narratives appear in the text referring to community participants? (e.g. report of experience, identification of problem with proposal, etc.)*  *How are these organised in relation to time and space (do they typically refer to immediate or long term outcomes? Do they concern themselves with different geographies?)* | Note the assumption of equivalence in the categories described above  The workshop as a whole is characterised as involving “interesting conversations…lively discussion…” [p9], however, the material emerging avoids any sense of conflict or enthusiasm which these terms suggest  Types of statement:  Future orientated statements make up the majority of statements reported as deriving from residents. Three categories of these are suggested: factual descriptions of (presumed) future states (“Access to fresh food in nearby facilities” [p10]), evaluate the impact of future organisation of space or events in relation to residents (“Difficulty in getting support activities” [p10]), or identify opportunities (“neighbours to help each other with transport, sharing costs, socialising” [p11])  However, is challenging to categorise these statements in semantic/grammatical terms as a wide range of different forms used with different possible interpretations of likelihood of occurring, particularly when chained together, with variation occurring even within sentences – e.g. contrast emphasised conditional clauses: “Smoking outside/smoking shelter **could potentially** create more litter which **will** need to be managed and **could** increase maintenance costs.” [p11, emphasis added] with lack of any verbs in this sentence: “Flexibility of the space within the development for activities, coffee clubs, floating support etc.” [p12] despite fact that both appear to refer to presumed future state and possibilities which may or may not come to pass. Note that statements typically identify anticipated outcome of current plan, rather than range of outcomes following different approaches/amendments  Other statement types include:  Request for information (“Need some clarity on service provision” [p10])  Statements linking personal knowledge/experience to specific problem/opportunity (“Topography can make walking around the area difficult with few benches to sit on” [p11])  Specific recommendation (“ensure that benches are spaced out regularly” [p11])  Few occasions in which different statements are chained together – e.g. statement on topography/benches above followed by:  “Recommendation to ensure that benches are spaced out regularly and appropriately - inaccessible areas without appropriate stopping points can create a feeling of failure” [p11]  “Steepness of hill leading into the development and in the general area. Difficulty in getting around by foot – this will range by fitness of the individual.  Walkability and extra benches – additional benches on the hill leading to the development would help. NCC Highways unlikely to accept responsibility to maintain additional benches/seating, covers to bus shelters also.  Recommendation – to query with NCC Highways. Active Travel Act and Welsh Government Walkability Questionnaire to form part of discussion.” [p16]  Typically focus only on the Estate and the immediate needs of residents, although are references to ‘wider community’ and how needs (e.g. mobility) will also be long term needs |
| At the level of semantics and grammar: how are statements presented in semantic and grammatical terms and what regularities and differences emerge?  *How are these statements framed in terms of:*  *language register (e.g. emotive / neutral)*  *grammatical features (e.g. declarative / conditional clauses; active/passive mood) semantic relations between clauses (e.g. separated or linked clauses)* | Very little variation in tone by different comments and recommendations  Almost all statements declarative rather than condition, whether present or future orientated |

|  | **Splott** |
| --- | --- |
| At the level of representing social events, how are different actors represented and brought into contact with each other?  *Which actors are present and which are absent?*  *How are these participants described?*  *Are there points of contact between individuals and/or groups described?*  *Are categories of equivalence and/or difference assumed, established or collapsed?* | Individual members of the HIA are named and there are initial descriptions of specific activities they carried out (e.g. who acted as facilitator to which group)  Viridor and Cardiff Council described as not being present – not clear what decisions were being made and how this would directly contribute to those decisions  The text reports discussion of possible tactics and outcomes in negotiations between the builder of the incinerator and Cardiff Council  ‘Vulnerable groups’ noted distinctly, but then apparently collapsed into a single category  A number of citations brought in to initial description of activities – this occurs both implicitly (the text notes evidence sources) and explicitly (the text describes specific interventions from facilitators to inform participants of academic evidence) |
| At the level of intertextuality, how do the voices of community/public participants ‘get in’ to the text?  *How is their speech translated into the text (e.g. direct reporting, indirect reporting, etc.)?*  *How does this orientate them in relation to difference and consensus?* | Text describing workshop presented as a narrative, with opinions and comments described in terms of specific speakers  Only one instance of direct quotation, used at end of section in a distinctly rhetorical way to connect the HIA directly with the legitimacy of the wider decision making processes. Notably this was a comment in the evaluation of the workshop rather than a comment during it:  “One of the stakeholders wrote that what they felt was most important was “Ensuring this community commitment influences the process…”” [p41]  Effect is of multiple voices, with consensus emerging only as a summary at the end of each section. Again, no description of how summary is developed. However, is also clear but unacknowledged that authorial role exists in organising a narrative to dramatize voices (“Yet another stakeholder wondered….” [p29]; “One stakeholder was quick to point out…” [p31]) |
| At the level of semantics and grammar: how does the use of language give or withhold agency from public/community participants?  *Are they predominantly described in the active or passive voice?*  *Are personal or impersonal forms used?* | Statements are typically presented in the active voice, although one consistent strategy is to present an initial statement in the active and subsequently switch to a passive voice to record additional comments or elaborations  There is a marked tendency to assign emotive language to participant statements:  “fears were expressed” [p29]  “there were concerns expressed” [p 31]  Contributions are often expanded over multiple clauses/sentences, often with qualifiers and modifiers (“Another stakeholder said that they were concerned…..This same individual also questioned…” |
|  |  |
| At the level of discourse*,* genre and intertextuality: How are statements and narratives organised and brought into relation with each other within the text?  *What genres are drawn on and what are the implications of these choices?*  *How is the text physically organised on the page and does this create specific discourses?*  *How is ‘evidence’ from community participants brought into contact with other forms of evidence (if at all)?* | As described above, the evidence is presented embedded within a narrative structure and uses language and rhetorical devices that serve to dramatize the emotional states and stakes of the participants:  “Another hope expressed by several stakeholders…. Fears were also expressed…” [p29]  There is a shifting of voice within the structure of the report that suggests the report is underlined by structures characteristic of narratives: authorial framing, nominally ‘transparent’ reporting of discussion and then authorial summary  It is noted at several points that participants express frustration due to lack of access to evidence: evidence is seen as held elsewhere  There is also a discourse of social justice in this HIA with participants’ ‘rational’ economic interest and beliefs/personhood described to validate and legitimate this narrative  “ [concerns] of the perceptions that outsiders have of Splott” [p32] |
| At the level of representation of social events: what categories are described/created within the text to define how participants present themselves/their evidence?  *What kinds of statements/narratives appear in the text referring to community participants? (e.g. report of experience, identification of problem with proposal, etc.)*  *How are these organised in relation to time and space (do they typically refer to immediate or long term outcomes? Do they concern themselves with different geographies?)* | The most frequently reported comments could be categorised as expressions of hope/concern for future developments expressed in uncertainty (“There was concern expressed…”/”One stakeholder said they felt unsure…”) [p 28]  However, there were a range of other types of statement, the majority of which might be classified under the following headings  A number of points where evidence from other sources is discussed: however, this does not appear to be integrated into recommendations or further commentary from authors or participants: “Another stakeholder said they were concerned that air pollution emissions figures submitted to the Environment Agency Wales from incinerators were currently based on average figures rather than continuously monitored real-time data. This, they felt, might mean that any short-term rises in the output of the plant’s emissions beyond agreed limits with the EAW would be missed.” [p32]  Commentary on motivations of other actors (“it was pointed out by one stakeholder that…developers are more likely to have the upper hand” [p 29]  Assertions based on values (“One stakeholder was quick to point out that incineration is a better solution….than…landfills” [p 31])  Commentary on specific plans and evidence (“This same individual also questioned the large scale of the facility…” [p32])  Novel ideas (“why not run a visitor centre?” [p36])  There are a small number of examples of ‘chaining’ of evidence, with this drawing/dependent on narrative structure, using ‘therefore’:  “There was therefore a recognition that more waste industry in the area will likely make these potentially negative perceptions of Splott harder to shift in the future. Two stakeholders therefore said that they felt that the environmental concerns highlighted in the workshop had the potential to negatively affect future investments in the community which would affect all groups” [p 32-33]  There is a notable engagement with the idea of long and short term outcomes  Splott enters the narrative as a site that has a distinct meaning for the community, with other places characterised as having negative views of the area. There is a sense of Splott as a character in itself, generally presented passively as somewhere that needs support from its community (e.g. “Splott might see its relative socio-economic disadvantage reinforced” [p 29]) |
| At the level of semantics and grammar: how are statements presented in semantic and grammatical terms and what regularities and differences emerge?  *How are these statements framed in terms of:*  *language register (e.g. emotive / neutral)*  *grammatical features (e.g. declarative / conditional clauses; active/passive mood) semantic relations between clauses (e.g. separated or linked clauses)* | Notable variation of tone across different statements, with more obvious rhetorical devices in evidence apparently related to the degree to which the statement was considered novel by the authors.  (“An interesting mitigation suggestion emerged from one of the stakeholders at this point: why not run a visitor centre?” [p36])  Possible implication that such statements, appearing without the structure of established narrative (e.g. without reference to previous discussion, without any apparent further comment from other participants that creates any narrative dynamic) the form struggles to integrate them |

|  | **Anglesey** |
| --- | --- |
| At the level of representing social events, how are different actors represented and brought into contact with each other?  *Which actors are present and which are absent?*  *How are these participants described?*  *Are there points of contact between individuals and/or groups described?*  *Are categories of equivalence and/or difference assumed, established or collapsed?* | Authors introduced by role/job function, but not by name  Opinions of stakeholders are consistently presented as unified. At some points wording is explicit that this was the case (“The workshop was unanimous…..” [p38]), but in most the participants are presented as having a collective opinion (“Stakeholders believed….” P39]  No explicit references to decision makers or processes |
| At the level of intertextuality, how do the voices of community/public participants ‘get in’ to the text?  *How is their speech translated into the text (e.g. direct reporting, indirect reporting, etc.)?*  *How does this orientate them in relation to difference and consensus?* | All speech is indirectly reported, although there is an instance of quotation from an industry representative interviewed in a trade magazine  Generally referred to as ‘stakeholders’: however, also a tendency for statements which may be assumed to come from participants to appear with no pronouns etc. identifying them as such (“Older people may be afraid to go for a walk….” p38] |
| At the level of semantics and grammar: how does the use of language give or withhold agency from public/community participants?  *Are they predominantly described in the active or passive voice?*  *Are personal or impersonal forms used?* | Generally positioned as active in grammatical terms, quite frequent assignment of relatively emotive/personalised words to described future orientation or current evidence provided: believed, felt, etc. |
|  |  |
| At the level of discourse*,* genre and intertextuality: How are statements and narratives organised and brought into relation with each other within the text?  *What genres are drawn on and what are the implications of these choices?*  *How is the text physically organised on the page and does this create specific discourses?*  *How is ‘evidence’ from community participants brought into contact with other forms of evidence (if at all)?* | Frequent use of references as in a peer–reviewed paper  Description and justification of selection / sampling methods  Tables used to organise perceptions of positive/negative factors of plans  Report structure used with multiple clearly labelled sections, shaded boxes for recommendations and structure of introduction, comments, summary. However, although the text is arranged (and language used) suggests narrative form, many paragraphs essentially function as lists of comments, rather than suggesting the strong narrative structure found in other reports  Authors actively bringing in different evidence: “The danger of driving dehydrated was discussed with each and their attention drawn to a recent article in CVDriver….” [p44]  Although there are clear sections, these do not always appear to define/contain the material around which they are nominally organised, e.g. [pp42-43] section ‘issues to consider from the focus groups’ appears first to continue presenting material, then shifts to describing a test of smartphone apps by the author |
| At the level of representation of social events: what categories are described/created within the text to define how participants present themselves/their evidence?  *What kinds of statements/narratives appear in the text referring to community participants? (e.g. report of experience, identification of problem with proposal, etc.)*  *How are these organised in relation to time and space (do they typically refer to immediate or long term outcomes? Do they concern themselves with different geographies?)* | Statements of presumed fact, often about current stakeholder motivations: “Workshop stakeholders believed that HGV, bus or coach drivers may be reluctant to drink sufficient fluids in order to reduce the need for toilet breaks” [p37]  Statements very focused in terms of time and place, but often broadened to consider other groups (e.g. tourists) that were not represented in the participant group  Most frequent statements were relatively straightforward predictions of future outcomes (“The workshop was unanimous in its view that reduced availability of facilities would not impact on a family’s choice to visit certain areas” [p38] and statements on current knowledge/ beliefs (“It was recognised that some residents living near public toilets may be hoping for closure….”  There were also a number of statements relating specifically to process and its ethics: “Stakeholders believed that shutting public toilets went against public policies…” [p39]  The relatively limited range of statement types may have been related to the specificity of this HIA or the limits of the workshop or some other reason  Very little evidence of ‘chaining together’ of evidence statements |
| At the level of semantics and grammar: how are statements presented in semantic and grammatical terms and what regularities and differences emerge?  *How are these statements framed in terms of:*  *language register (e.g. emotive / neutral)*  *grammatical features (e.g. declarative / conditional clauses; active/passive mood) semantic relations between clauses (e.g. separated or linked clauses)* | There are a number of points at which the language register suggests a blurring of genres and of distinctions between voices: “Some of the conveniences have been converted into much needed homes…” [p38]  Very little variation in language register |

|  | **Ffos-Y-Fran** |
| --- | --- |
| At the level of representing social events, how are different actors represented and brought into contact with each other?  *Which actors are present and which are absent?*  *How are these participants described?*  *Are there points of contact between individuals and/or groups described?*  *Are categories of equivalence and/or difference assumed, established or collapsed?* | Decision makers are noted at the beginning of the report, with attempts to engage them; also some of those involved in decision making (e.g. local councillors) are noted as ‘being involved’ – however, not positioned as such in the text; the processes and personnel of decision making post report are absent from the body of the text  Often use of general “One resident wanted to know why the authorities decided on this scheme” [p56]  Where actions deriving from entities are mentioned, these are often in terms of the project rather than the body “The Ffos-Y-Fran scheme will remove a further 900 acres of the remaining urban common land from the mountainside” [p65]  Residents and local political representatives also appear to have been collapsed into a single category  Participants described as ‘citizens’ at outset, but not then used  A number of distinctions within the resident community, notably gypsy/traveller community [p23-24], older people [p62] and young people [p61], whose needs are described specifically, with young people given voice |
| At the level of intertextuality, how do the voices of community/public participants ‘get in’ to the text?  *How is their speech translated into the text (e.g. direct reporting, indirect reporting, etc.)?*  *How does this orientate them in relation to difference and consensus?* | Considerable use of direct quotation throughout the document  Often this precedes or follows summaries of perspectives from those who have contributed to the workshop. These typically fall into one of four categories, with the first two being most common:  1.simple factual statements that appear to be introduced to add legitimacy to a point just made:  “Residents point out that the proposed open-cast is likely to have a negative impact on inward investment, especially in attracting clean industry. As one resident stated:  “There would be inward investment if the scheme did not go ahead”” [p53]  2. comments that demonstrate strong feeling related to specific points being made:  “May I express as a local and very near resident to the proposed site my utter disgust  and dismay that once again the health of the people of Merthyr Tydfil is being sacrificed for financial gain (resident letter dated 31/05/06).” [p57]  3. specific lived experiences – there are fewer of these related to current project, but several drawn in from other projects: “The proposed Phase IIIa Ffos-Y-Fran opencast is very close to my property, about 250 metres away, we will have another life of hell, but this time it will be for 22  years, there are toxic waste tips being dug up to get at the coal, this will mix with coal  dust and diesel fumes and pollute the air all around us and Mountain Hare (Resident  letter dated 15/06/06)” [p40]  4. Concerns about the future: “My concern is particularly about the effects on chest conditions…” [p32] |
| At the level of semantics and grammar: how does the use of language give or withhold agency from public/community participants?  *Are they predominantly described in the active or passive voice?*  *Are personal or impersonal forms used?* | There are a number of instances in which intensifiers are used to attach ideas more clearly to residents – e.g. “Residents firmly believed…” [p66]  Residents generally presented in ‘active’ voice |
|  |  |
| At the level of discourse*,* genre and intertextuality: How are statements and narratives organised and brought into relation with each other within the text?  *What genres are drawn on and what are the implications of these choices?*  *How is the text physically organised on the page and does this create specific discourses?*  *How is ‘evidence’ from community participants brought into contact with other forms of evidence (if at all)?* | Evidence from report on Margam site is brought in regularly, often to counterpoint the evidence provided by participants in this HIA  Methods section and rigorous citation practices followed throughout  At a number of points (e.g. p18) resident comments are cited in an academic format – the academic genre apparently subsuming narrative reporting  Evidence from the workshop included in a wider category of evidence collected by local people, including newspaper reports, correspondence over time, etc.  Wider range of technical evidence used, including plans and photos  Evidence of interplay of a number of genres, including narrative, e.g. pp52-54, in which a number of stakeholder voices are brought into contact to drive forward a description of perceived advantages and disadvantages of the development  Frequent mixture of published peer-review evidence, reports from previous health impact assessments  Participants also bring their own evidence: pp18-19, for example, report evidence submitted by a resident that has processed existing published sources into an evidence synthesis: some of this is further organised and formatted by the authors into tables (e.g. p19)  In other places, residents (who do include individuals such as former miners who might have specific knowledge in this area) provide evidence which is simply presented in the text with no comment from the authors on verification – e.g. “One resident stated that coal dust goes straight into lungs, and would especially affect children for the rest of their lives” [p31]. In some cases, further evidence is put forward (e.g. p32) – again, adduced solely in support of the resident perspective – it is not clear how the evidence provided in support was produced nor what levels of uncertainty may surround it |
| At the level of representation of social events: what categories are described/created within the text to define how participants present themselves/their evidence?  *What kinds of statements/narratives appear in the text referring to community participants? (e.g. report of experience, identification of problem with proposal, etc.)*  *How are these organised in relation to time and space (do they typically refer to immediate or long term outcomes? Do they concern themselves with different geographies?)* | Use of other sources of ‘public generated’ evidence (e.g. newspapers and correspondence) produces a wider timespan of perspective  Considerable attention from residents into longer term issues, particularly around decisions on housing and settling in the area - notable that the one category specifically defined within the wider category of residents is young people, who repeatedly adopt a longer term perspective to (at least in part) legitimise their points of view  Statements of current fact and specific outcomes made up the majority of statement types, but there were multiple examples of other categories:  Statements of current (perceived) fact: “Residents living close to Ffos-Y-Fran and who attended the workshop stated that there are more than 60 planning conditions” [p38]  Statements about outcomes of the specific plan: “Young people believed that the scheme would take away the history of the area…” [p62]  Statements about the past, lived experience: “One resident also describes how the dust suppression measures promised during a housing development in the area never materialised” [p39]  Commentary on the motivations of other actors: “Residents attending the workshop found it ironic that Merthyr Tydfil was reintroducing mining after all the pit closures of the 1980s” [p57]  Statements of values: “Residents…. mentioned that the scheme was putting ‘profits before people’” [p57]  Requests for information: “Residents attending the workshop wanted to know who guided the developer towards tips….” [p75]  Much more chaining of evidence together – e.g. section on dust deposit [pp36-37] has following sequence:   1. Statement that residents believe there will be a great deal of dust created through movement of earth to create the opencast mining site 2. Supporting evidence from Air Quality Statement provided 3. Evidence of potential harms from academic literature discussed 4. Resident perceptions of amount of dust that would be created in more specific terms 5. Further project-specific evidence produced on the amount of dust created 6. Further academic literature on harms exhibited in animals cited 7. Resident beliefs about the harms this would create to their health   However, it is not clear what the resident-derived evidence here adds to the literature  Issues of integrating evidence around opportunities into the text |
| At the level of semantics and grammar: how are statements presented in semantic and grammatical terms and what regularities and differences emerge?  *How are these statements framed in terms of:*  *language register (e.g. emotive / neutral)*  *grammatical features (e.g. declarative / conditional clauses; active/passive mood) semantic relations between clauses (e.g. separated or linked clauses)* | Considerable use of ‘felt’ and ‘believe’ – however, also multiple instances in which greater certainty appears to be given to resident commentary on future outcomes – e.g.  “Residents state that even though dust will be damped down, there will still be dust falling on properties” [p39]  Markers in the text that residents challenge the evidence presented by statutory bodies: e.g. use of quotation marks in “Younger people attending the workshop expressed concerns about the way ‘official’ documents have reported the effects of the wind direction in the area” [p43] |

|  | **WALLS Scheme** |
| --- | --- |
| At the level of representing social events, how are different actors represented and brought into contact with each other?  *Which actors are present and which are absent?*  *How are these participants described?*  *Are there points of contact between individuals and/or groups described?*  *Are categories of equivalence and/or difference assumed, established or collapsed?* | Steering group with overall guidance of the project is listed by name and affiliation, although is noted that not all were present at every meeting. Authorship of specific document not mentioned  Different mixes of groups invited to different specific meetings, including focus groups. Reports from these are brought together in a single section [7 and 8], different categories of individual (e.g. landlords and tenant) appear to be separated into different paragraphs  Reference to ‘citizen power’ – positioning of those taking part as at least having visibility of selves as citizens  No reference to ‘decision makers’ or decision making process |
| At the level of intertextuality, how do the voices of community/public participants ‘get in’ to the text?  *How is their speech translated into the text (e.g. direct reporting, indirect reporting, etc.)?*  *How does this orientate them in relation to difference and consensus?* | Early in the text, key findings note that there was no consensus in a number of key areas [p7]  No direct quotation: majority of speech is reported, although there are instances of ‘competing voices’, with dissenting comments noted (e.g. p33) and instances in which a single member is associated with a particular point of view |
| At the level of semantics and grammar: how does the use of language give or withhold agency from public/community participants?  *Are they predominantly described in the active or passive voice?*  *Are personal or impersonal forms used?* | Participants typically presented as subject of sentences that involve them, sentence structure typically very simple, declarative form |
|  |  |
| At the level of discourse*,* genre and intertextuality: How are statements and narratives organised and brought into relation with each other within the text?  *What genres are drawn on and what are the implications of these choices?*  *How is the text physically organised on the page and does this create specific discourses?*  *How is ‘evidence’ from community participants brought into contact with other forms of evidence (if at all)?* | Key findings at the beginning present all identified impacts as a list, with no suggested internal structure  Genre: presented as an ‘official report’, with numbered paragraphs throughout and executive summary at the beginning. |
| At the level of representation of social events: what categories are described/created within the text to define how participants present themselves/their evidence?  *What kinds of statements/narratives appear in the text referring to community participants? (e.g. report of experience, identification of problem with proposal, etc.)*  *How are these organised in relation to time and space (do they typically refer to immediate or long term outcomes? Do they concern themselves with different geographies?)* | Makes considerable efforts to caveat the usefulness of opinions:  “As number of opinions were expressed….as to the potential benefits and dis-benefits of the scheme….as predictions, these opinions could only be speculative…. [p32]  There is a ‘bracketing off’ of evidence again  The structure of the text presents projections about the future as being comparable, even when there are a number that look to substantially wider horizons than the majority – e.g. p34, technical concerns about tenancy rights in individual cases are presented in paragraphs in the same way as comments about possibilities of substantial changes to the housing market  Types of statement: the majority of statements either concerns proposed outcomes, or suggestions for improving the plan. There was a notably high proportion of the latter in the text compared with other reports  Statements about outcomes of the specific plan: “It was felt that the proposed scheme would ensure that landlords would gain a better knowledge of the rights and entitlements” [p33]  Note that this also included statements specifically noted as being counter-statements: “A number of counter arguments against the above points were also posed” [p34]  Suggestions to improve the scheme: “Workshop participants also suggested that support agencies with understanding of the needs of tenants’ needs [sic]….should be involved in training…” [p35]  Commentary on motivations of other actors: “A workshop participant felt there was a danger that some organisations…would try to mislead landlords…” [p35] |
| At the level of semantics and grammar: how are statements presented in semantic and grammatical terms and what regularities and differences emerge?  *How are these statements framed in terms of:*  *language register (e.g. emotive / neutral)*  *grammatical features (e.g. declarative / conditional clauses; active/passive mood) semantic relations between clauses (e.g. separated or linked clauses)* | Considerable use of conditional constructions to ‘double hedge’ predictive comments: “Some [participants] felt that in order to avoid licensing, landlords might leave their properties empty…” [p33]  Note the emotional register is comparatively neutral: verbs such as ‘felt’ are rare relative to more neutral words such as ‘suggested’ |

|  | **Cam Ymlaen** |
| --- | --- |
| At the level of representing social events, how are different actors represented and brought into contact with each other?  *Which actors are present and which are absent?*  *How are these participants described?*  *Are there points of contact between individuals and/or groups described?*  *Are categories of equivalence and/or difference assumed, established or collapsed?* | Authors and affiliations named at beginning of report, not thereafter  No distinction between service users: all as a single group  Participants’ (Service users) individual comments evaluating process presented in appendix  Cam Ymlaen becomes entity in itself and is counterpointed to other entities which fail to understand the benefits it provides to them - through support for service users is assumed, although often unstated – therefore service users in fact are (Self?) edited out of these relationships (“Cam Ymlaen can help save the Government money”)  Other entities mentioned within recommendations for each section: Welsh Govt (target for lobbying); service user and story (for use in lobbying); individual within Council who might offer practical support. Note that in recommendation sections, other entities are described with more detail/specificity (e.g. “Welsh Assembly Government” in Recommendations vs “Welsh Government” in main text. Raises issues of whose voice / how managed we are hearing – do the recommendations incorporate voice of report authors? |
| At the level of intertextuality, how do the voices of community/public participants ‘get in’ to the text?  *How is their speech translated into the text (e.g. direct reporting, indirect reporting, etc.)?*  *How does this orientate them in relation to difference and consensus?* | Generally presented as short notes (similar to Gaer in style, organisation and genre). As with that report, all statements presented as consensus of single entity of ‘service users’  A number of instances in which specific words of participants are presented (“Cam Ymlaen “wakes you up a bit!”” p3)  Also a number of instances where textual markers are used (it appears) as rhetorical device to bring reader closer to voices of service users – e.g. quotation marks - “”Normalises” issues” [p4] |
| At the level of semantics and grammar: how does the use of language give or withhold agency from public/community participants?  *Are they predominantly described in the active or passive voice?*  *Are personal or impersonal forms used?* | Similar to Gaer report – often service users do not appear either as subject or object of sentences |
|  |  |
| At the level of discourse*,* genre and intertextuality: How are statements and narratives organised and brought into relation with each other within the text?  *What genres are drawn on and what are the implications of these choices?*  *How is the text physically organised on the page and does this create specific discourses?*  *How is ‘evidence’ from community participants brought into contact with other forms of evidence (if at all)?* | Very similar to Gaer in style, organisation and genre: short notes, organised into positive/negative lists using different categories (drawing on Dahlgren and Whitehead’s model) to organise material |
| At the level of representation of social events: what categories are described/created within the text to define how participants present themselves/their evidence?  *What kinds of statements/narratives appear in the text referring to community participants? (e.g. report of experience, identification of problem with proposal, etc.)*  *How are these organised in relation to time and space (do they typically refer to immediate or long term outcomes? Do they concern themselves with different geographies?)* | Notable that, in comparison with other reports using tables and short notes, there appears to be a strong distinction between the two sides. They are dissimilar in terms of the amount of text; they are not strongly linked by parallel points and they are characterised by statements of perceived fact (“Leads to positive role models” [p4]) and suggestions for activities (“Go out to employers to dispel myths” [p4]) on the positive side and statements expressing uncertainty on the negative side (“Hard to quantify” [p6]) on the negative side  There is a noticeable orientation in this report around actions that can be taken (it is assumed by the participants/service users) to address the issues raised:  “Lobby Welsh Assembly Government….” [p5]  Very little evidence of any chaining together of evidence |
| At the level of semantics and grammar: how are statements presented in semantic and grammatical terms and what regularities and differences emerge?  *How are these statements framed in terms of:*  *language register (e.g. emotive / neutral)*  *grammatical features (e.g. declarative / conditional clauses; active/passive mood) semantic relations between clauses (e.g. separated or linked clauses)* | Very neutral voice throughout, although as noted above, instances in which textual conventions (e.g. quotation marks) are used apparently to ‘bring through’ the voices of service users  “Leads to long term impact on community through contact with people with mental illness. “Normalises” issues” [p4] |

|  | **Llangefni** |
| --- | --- |
| At the level of representing social events, how are different actors represented and brought into contact with each other?  *Which actors are present and which are absent?*  *How are these participants described?*  *Are there points of contact between individuals and/or groups described?*  *Are categories of equivalence and/or difference assumed, established or collapsed?* | Developer noted in text as making a presentation  Stakeholders presented as a single group  “The Planning process was seen as having a mildly positive impact on citizen power and influence” [p46] – one of a number of references to ‘citizens’ – however, seems to separate/ compartmentalise citizenry from participation?  Notable that “the public” are mentioned as (subsequent to workshop) having commented on the processes around the HIA and planning permission, but this commentary is mentioned but not integrated into text in relation to participant discussions (also see intertextuality and genre sections below) |
| At the level of intertextuality, how do the voices of community/public participants ‘get in’ to the text?  *How is their speech translated into the text (e.g. direct reporting, indirect reporting, etc.)?*  *How does this orientate them in relation to difference and consensus?* | Strong tendency to present comments as consensus derived from single participant opinion  Decision making processes mentioned as described above, in context of public commentary subsequent to workshop on processes – notable that this is mentioned, but cannot (it appears) be integrated into the text of the workshop directly but effectively appears as an addendum |
| At the level of semantics and grammar: how does the use of language give or withhold agency from public/community participants?  *Are they predominantly described in the active or passive voice?*  *Are personal or impersonal forms used?* | Strong tendency to use passive tense when referring to comments of participants |
|  |  |
| At the level of discourse*,* genre and intertextuality: How are statements and narratives organised and brought into relation with each other within the text?  *What genres are drawn on and what are the implications of these choices?*  *How is the text physically organised on the page and does this create specific discourses?*  *How is ‘evidence’ from community participants brought into contact with other forms of evidence (if at all)?* | Overall, the style of writing (and of visual presentation) appears to be very narrative driven – however, there are notable differences to other narrative styles (particularly in the lack of use of voices and discussion to create any recognisable narrative structure) and similarities to more note-based, report-type HIAs – e.g. sentences tend to present simple positives and negatives, with no additional context or rooting in social exchange  First description within the participatory workshop is of presentation by developer, therefore evidence from this source brought directly into contact with participants  Notable that the authors seem to disappear in this section as participants, and only exist as observers: they are interpreting on the semi-explicit basis of observation, with notably tentative language being used:  “It is possible that the suggestion of training overcame any of the stakeholder’s concerns that the local workforce may not have the skills to apply for the jobs being provided. There does appear to be a recommendation that local training establishments would need to become more involved” [p45]  Notable at other points that author(s) do not address apparent error in technical understanding by participants, but present their alternate interpretation in the text  Scoring and use of scores to develop table aligning all comments on ‘positive/negative’ axis; again implication is that this will be used to drive decision making |
| At the level of representation of social events: what categories are described/created within the text to define how participants present themselves/their evidence?  *What kinds of statements/narratives appear in the text referring to community participants? (e.g. report of experience, identification of problem with proposal, etc.)*  *How are these organised in relation to time and space (do they typically refer to immediate or long term outcomes? Do they concern themselves with different geographies?)* | Statements are almost entirely limited to comments on outcomes of the proposals: “They felt that improving income levels in the area would have a very positive impact on family life and diet” [p45]  Typically very limited in scope: highly focused on immediate/short term outcomes limited to local area – major exception is the comment from the developer on the benefits of increasing renewable energy provision in the UK, which is noted as receiving positive response from participants |
| At the level of semantics and grammar: how are statements presented in semantic and grammatical terms and what regularities and differences emerge?  *How are these statements framed in terms of:*  *language register (e.g. emotive / neutral)*  *grammatical features (e.g. declarative / conditional clauses; active/passive mood) semantic relations between clauses (e.g. separated or linked clauses)* | Language register is strongly neutral throughout with consistent use of distancing forms of grammar (e.g. using third person)  Most/all of text presented in consistent language register |
